# Public accountability and health policy: the case of the 60-day Law in ensuring access to breast cancer treatment in São Paulo State

**DOI:** 10.31744/einstein_journal/2026AO2286

**Published:** 2026-05-21

**Authors:** Fernanda Borges Keid, Nivaldo Carneiro, Vânia Barbosa do Nascimento

**Affiliations:** 1 Centro Universitário FMABC Postgraduate Program Santo André SP Brazil Postgraduate Program, Centro Universitário FMABC, Santo André, SP, Brazil.; 2 Centro Universitário FMABC Department of Collective Health Santo André SP Brazil Department of Collective Health, Centro Universitário FMABC, Santo André, SP, Brazil.

**Keywords:** Breast neoplasms, Health policy, Health care quality, access, and evaluation, Health equity, Public accountability

## Abstract

**Objective:**

This article analyzed the impact of Law No. 12,732/2012 on breast cancer care in São Paulo State (Brazil) between 2013 and 2024, as well as the role of external oversight.

**Methods:**

This qualitative-quantitative study used 58,892 records from the São Paulo Oncocenter Foundation database and cases judged by the São Paulo State Court of Accounts.

**Results:**

The analysis revealed significant and persistent delays in diagnosis (31.3-66.8%) and treatment initiation (38-72%) across all Regional Health Directorates, although delays fluctuated over time, no consistent trend toward reduced delays was observed between the 2013-2024 period. The São Paulo State Court of Accounts, as an external oversight mechanism, focused on formal analyses rather than results-oriented evaluations, particularly regarding the identified delays.

**Conclusion:**

Our findings led us to conclude that the 60-day Law has had a limited impact in ensuring timely access to breast cancer diagnosis and treatment in the state, as delays have remained high and have not consistently improved over time. It is recommended that external control strategically strengthen this public policy by inducing managerial accountability and ensuring the right to access and comprehensive care.

## In Brief

The 60-day Law has limited impact in São Paulo. Persistent delays in breast cancer diagnosis and treatment (2013-2024), with no consistent improvement. External oversight, focused on formality, does not directly evaluate results. Reorienting oversight is recommended to induce managerial accountability and ensure dignified access.

## Highlights

■ Law 12.732/12 had limited impact on breast cancer treatment access.
■ Persistent delays in breast cancer diagnosis & treatment in São Paulo, 2013-2024.
■ External oversight prioritizes compliance, not direct results evaluation.
■ Reorient oversight to induce greater managerial accountability.

## Graphical abstract

**Figure:**
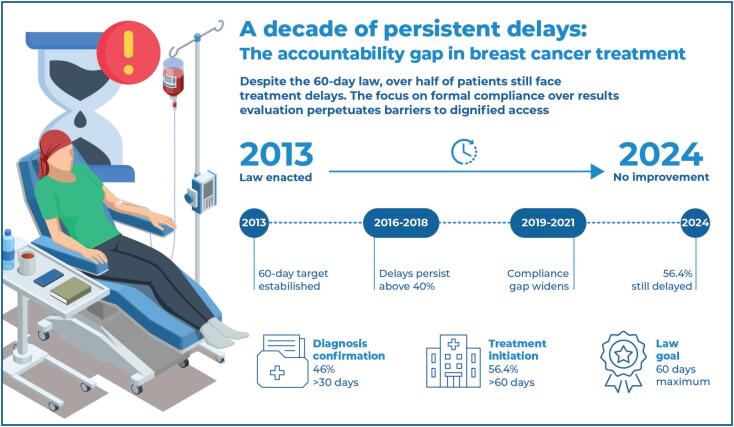

## INTRODUCTION

Breast cancer is considered one of the most impactful malignancies on global public health, being the most prevalent among women. The situation is exacerbated by high mortality rates, which, in 2018 alone, resulted in 17,572 deaths in Brazil, accounting for 16.4% of total cancer-related deaths.^(1)^ In the 2020 Oncology State Plan of São Paulo, cancer was the second leading cause of mortality in 2019 (130.33/100,000 inhabitants), with breast cancer being the primary cause of cancer deaths among women.^(2)^

This reality underscores the urgency in addressing this disease. Delays in diagnosis and treatment initiation significantly reduce cure chances, reinforcing the principle that swift therapeutic intervention is crucial for a favorable prognosis.^(3,4)^

In response to this need and in an effort to mitigate inequities in access to cancer care, Law No. 12.732/12, popularly known as the 60-day Law, was enacted to establish a maximum timeframe to begin treatment after confirmed malignant neoplasms from the date of diagnosis.^(5,6)^

In Brazil, oncological care policy within the Brazilian Public Health System (SUS – *Sistema Único de Saúde*) has been formulated and consolidated through a regulatory framework aimed at ensuring the constitutional right to health. This right, enshrined by the 1988 Federal Constitution and regulated by Law No. 8.080/1990, was strengthened with the publication of the SUS Users’ Rights Charter in 2009. Specifically for cancer patients, regulations such as the National Policy for Cancer Prevention and Control (Law No. 12.732/2012), which establishes a 60-day timeframe for treatment initiation, and Law No. 13.896/2019, which sets a 30-day timeframe for confirmatory tests, stand out as key tools in comprehensive care and minimizing socioeconomic disparities in service access.^(7)^

In São Paulo State, cancer care in SUS is integrated into a complex and multifaceted network with 17 Regional Health Directories (DRS - *Departamentos Regionais de Saúde*), integrated into Regional Health Care Networks, which function as administrative units of the State Health Department, responsible for managing and coordinating services with Regional Oncology Plans developed and ratified to organize care. The Hebe Camargo Cancer Combat Network, created by State Decree No. 62.394/2016, coordinates a set of diagnostic and treatment service units authorized by the Brazilian Ministry of Health.^(2,8)^ The Central Regulation of Health Service Offers (CROSS) plays a central role in managing access and patient allocation.^(2)^ The complexity of this network is further heightened by the increasing involvement of Health Social Organizations (OSS - *Organizações Sociais de Saúde*) in managing various public health services,^(9)^ including the operation of vacancy provision by CROSS itself.

Despite this robust regulatory framework and intricate care network, the implementation of Law No. 12.732/2012 in São Paulo has posed challenges. Data from the National Cancer Institute (INCA - *Instituto Nacional de Câncer*) has shown that, in 2019, 45.22% of cancer cases in São Paulo lacked treatment information, and only 28.84% began treatment within 30 days,^(2)^ highlighting not only users’ difficulty in accessing cancer care but also a critical legal gap: the 60-day Law does not establish direct and automatic administrative sanctions for public entities that fail to comply with its provisions, which risks undermining its normative force.^(6,10)^

The state's effectiveness in meeting social needs requires continuous improvement in the development, execution, and evaluation of public policies, aiming to optimize resource application. Government actions, aligned with the fundamental objectives of Article 12 of the 1988 Constitution, demand rigorous oversight, conducted both by institutions responsible for policies and by external bodies.^(11)^ Therefore, the role of external oversight becomes essential to ensure compliance with health legislation. The São Paulo State Court of Accounts (TCESP - *Tribunal de Contas do Estado de São Paulo*) is responsible for supervising the management of public resources,^(9,12)^ acting as a guarantor of good governance principles and a promoter of public accountability, a concept involving the role of external oversight in the implementation processes of public health policies, transparency, and managerial accountability to society.^(13,14)^ Hence, the TCESP conducts contract analyses, audits, and inspections to identify discrepancies and inefficiencies in public policies, resource allocation, and service delivery to society.

## OBJECTIVE

Given this context, we aimed to scrutinize the impact of Law No. 12.732/12, from 2013 to 2024, in ensuring timely access to breast cancer diagnosis and treatment in São Paulo State and analyze how administrative accountability mechanisms can enhance the effectiveness of legal norms and oncological public policies. Additionally, we sought to highlight the strategic role of the São Paulo State Court of Accounts and propose mechanisms for external oversight in the state to more effectively induce accountability and improve outcomes, thereby realizing effective rights for patients.

## METHODS

### Study design

We employed a qualitative-quantitative methodological approach, which focuses on the planned integration of different techniques and procedures to deepen our understanding of the investigated reality. Evaluating health programs and policy strategies using qualitative-quantitative methods can integrate the objective and subjective dimensions of reality by linking epidemiological and statistical indicators with narratives, practices, and meanings from social actors.^(15)^

This study was conducted in two complementary stages: a quantitative stage analyzing the time required to access breast cancer diagnosis and treatment, and a qualitative stage evaluating TCESP decisions. In the first stage, we conducted an ecological study using secondary data from 2013 to 2024 to investigate population patterns and trends. The study population comprised female patients aged ≥18, diagnosed with breast cancer (ICD-10 C50), and treated within the SUS network in São Paulo State.

### Data collection

Data were obtained from the Cancer Hospital Registry Database, coordinated by the Oncocentro Foundation of São Paulo (FOSP - *Fundação Oncocentro de São Paulo*),^(16)^ a public institution linked to the São Paulo State Health Department. Data collection was performed on July 15, 2025, using publicly available data from FOSP, resulting in a sample of 58,892 records. Temporal analysis was segmented into three-year periods (2013-2015, 2016-2018, 2019-2021, and 2022-2024) to enable a detailed assessment of the investigated phenomena over time.

We collected two dependent variables, with a primary focus on the time to diagnosis and treatment, according to the literature.^(17)^ The first was the time to diagnosis (measured in days), representing the interval between the first consultation and diagnostic confirmation. This variable was analyzed in both its continuous form and as a binary categorization (≤30 days vs. >30 days), adhering to the deadline established by Law No. 13,896/2019. The second variable, time to the start of treatment (measured in days), corresponds to the interval between diagnostic confirmation and the commencement of the treatment. It was analyzed as both a continuous variable and a categorized variable (≤60 days *versus* >60 days), in accordance with Law No. 12,732/12.^(5)^

The qualitative stage consisted of a documentary and hermeneutic analysis of the decisions rendered by the TCESP for the same period. Documentary analysis is a fundamental research technique as it enables access to temporal, institutional, and normative information that allows both explicit content and implicit meanings in official texts to be analyzed.^(18)^ Beyond formal description, a hermeneutic approach was adopted, as its interpretative characteristic enables one to assign meaning to texts within their production and application contexts. When applied to research in public health and public policies, hermeneutics contribute to understanding the actors’ intentionality, institutional tensions, and the value orientations in the analyzed documents.^(15)^

The analysis identified not only the normative and procedural dimensions of inspections but also the interpretations of the effectiveness of Law No. 12,732/12 within the scope of public health policy. Although hermeneutics can reveal hidden discourses and contexts, it has limitations when addressing the technical objectivity, measurement needs, and verifiability required by normative and management documents. These limitations make hermeneutics more effective when combined with systematic documentary analysis and quantitative and qualitative methods. This combination allows for a comprehensive understanding of the intervention's effectiveness, its limitations, and potential.^(15)^

Therefore, this stage aimed to critically interpret the Court of Accounts’ stance on regulating access to health, contracting in oncology, and above all, compliance with Law No. 12,732/2012. Data collection was conducted through a case law search in TCESP's website (https://www.tce.sp.gov.br/jurisprudencia/). Keywords and combinations related to the oncological care policy of São Paulo State and present in the official 020 Oncology State Plan were used. Temporal filters were applied from 01/01/2010 (creation date of CROSS), resulting in the following records: "*cross*" (n=794); "*rede hebe camargo*" (n=5); "*assistência oncológica*" (n=2); "*contrato de gestão*," AND "*cross*" (n=369); "*contrato de gestão*" AND "*rede hebe camargo*" (n=5); "*contrato de gestão*," "*acesso à saúde*," AND "*cross*" (n=5). From this initial pool of records, only those categorized as "report/vote" type documents were selected, as they effectively materialize the collegiate understanding of the TCESP on the analyzed matters. This filtering process resulted in 186 decisive acts being included documentary and hermeneutic analysis. The selected documents were subjected to a critical reading guided by the decision analysis method of Freitas et al,^(19)^ which allows one to identify narrative elements, decision-makers’ arguments, gaps in supervision, and opportunities for a more incisive action of external control in ensuring the effectiveness of public health policies.

### Statistical analysis

The statistical analyses were conducted using the R software (v. 4.4.2)^(20)^ and Statistical Package for the Social Sciences Software (v. 27.0).^(21)^ Variables were described as absolute frequencies (n) and relative percentages (%), and descriptive data were presented for the entire sample and additionally detailed by DRS and each aforementioned three-year periods, allowing for the identification of regional and temporal patterns.

## RESULTS

The 58,892 records analyzed, covering 2013–2024 and sourced from the FOSP database,^(16)^ formed the basis for analyzing variables aimed at understanding compliance with the deadlines established by the 60-day Law. The sample distribution by DRS and the percentages of delays for diagnosis and treatment for each region are detailed in table 1. Access time revealed significant challenges in complying with Law No. 12,732/2012 for the entire sample. Of the total, 46% of the cases presented delays in diagnosis and the initiation of treatment, and 56.4% of the treatments started late, with times exceeding those stipulated by the aforementioned legislation.

**Table 1:** Number of women diagnosed with breast cancer in the Brazilian Unified Health System in São Paulo State by Regional Health Directorate, according to diagnosis and treatment time, from 2013 to 2024

DRS	City	n (%)	Diagnosis ≥30 days (%)	Treatment initiation ≥60 days (%)
01	São Paulo (metropolitan area)	25679 (43.6)	41.6	55.8
02	Araçatuba	1140 (1.9)	55.9	67.5
03	Araraquara	855 (1.5)	36.6	45.6
04	Baixada Santista	2510 (4.3)	52.2	55.6
05	Barretos	1631 (2.8)	19.8	64.7
06	Bauru	2657 (4.5)	31.3	38.0
07	Campinas	6711 (11.4)	59.2	58.2
08	Franca	667 (1.1)	45.3	65.1
09	Marília	1754 (3.0)	41.1	41.4
10	Piracicaba	2408 (4.1)	55.9	54.4
11	Presidente Prudente	1447 (2.5)	66.8	58.7
12	Registro	171 (0.3)	63.7	49.1
13	Ribeirão Preto	2926 (5.0)	42.6	59.5
14	São João da Boa Vista	1491 (2.5)	64.6	72.0
15	São José do Rio Preto	3432 (5.8)	35.9	61.2
16	Sorocaba	1313 (2.2)	60.9	64.5
17	Taubaté	2100 (3.6)	61.9	55.4
**Total**		58892	46	56.4

Source: São Paulo Oncocenter Foundation Database. Prepared by the authors, 2025. DRS: Regional Health Directorate.

**Figure 1:**
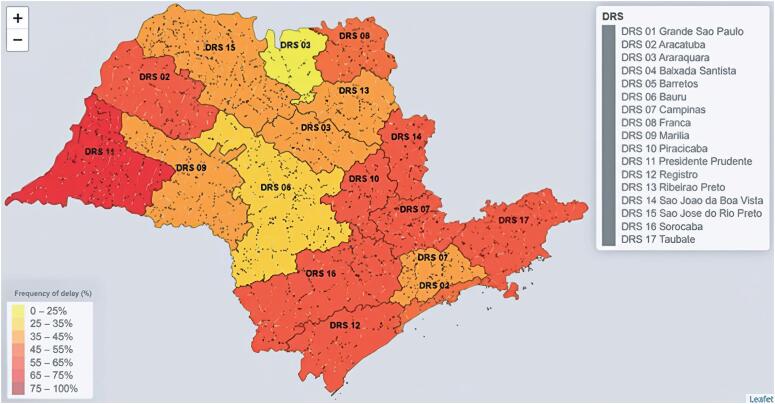
Frequency distribution of diagnostic delay (2013-2024)

**Figure 2:**
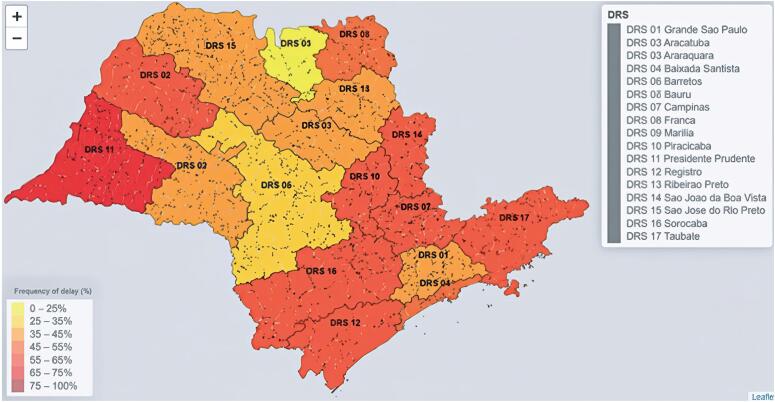
Frequency distribution of treatment delay (2013-2024)

Significant regional disparities were observed in meeting these deadlines (Table 1; Figures 1 and 2). DRS05 (Barretos), represented exclusively by *Hospital do Amor* (Fundação Pio XII), recorded the lowest rate of delays in the diagnosis (19.8%), indicating a greater adherence to the legal deadline at this stage. Conversely, DRS14 (São João da Boa Vista) had the highest treatment delays (72.0%), highlighting a significant non-compliance with the 60-day Law. DRS06 (Bauru) showed relative consistency (38.0%) in its delay rates for diagnosis and treatment, maintaining them comparably lower compared to other regions. This phenomenon could potentially be explained by the presence of a major oncology-specialized hospital, Hospital Amaral Carvalho, which is the regional reference center.

DRS: Regional Health Directorate.

Source: São Paulo Oncocenter Foundation Database. Prepared by the authors, 2025.

DRS: Regional Health Directorate.

Source: São Paulo Oncocenter Foundation Database. Prepared by the authors, 2025.

**Figure 3:**
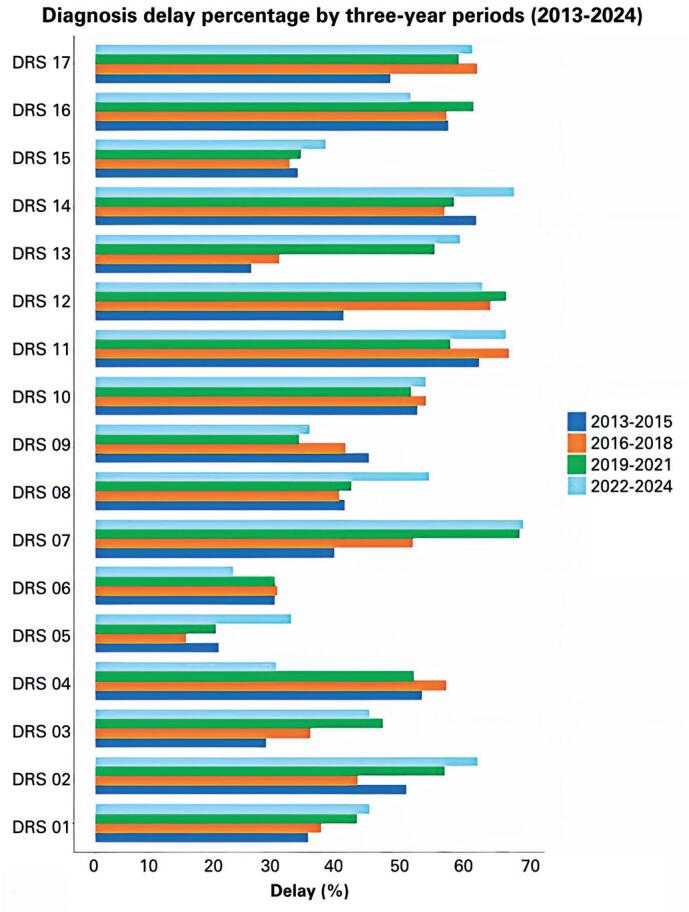
Percentage distribution of diagnostic delay by three-year periods (2013-2024)

**Figure 4:**
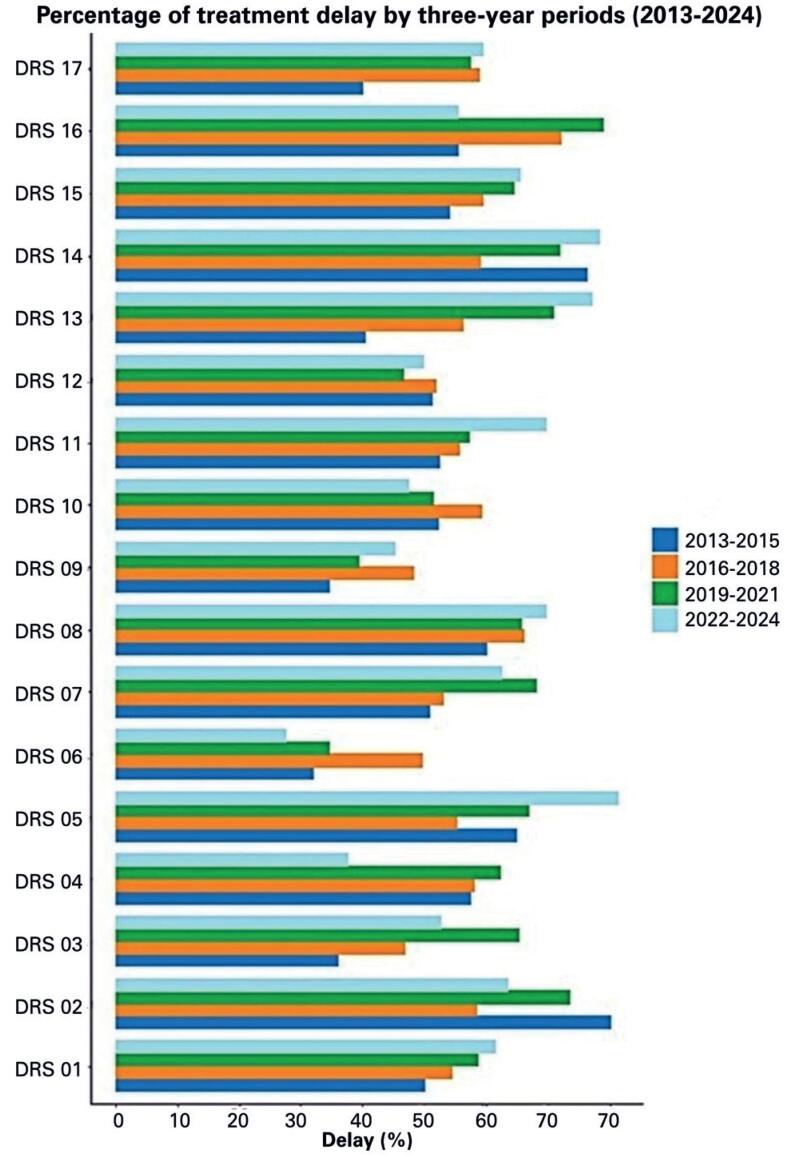
Percentage distribution of treatment delay by three-year periods (2013-2024)

A triennial analysis of the frequency distribution concerning diagnosis and treatment initiation delays was conducted to facilitate a detailed observation of the evolution and persistence of these challenges. This approach allowed for the identification of whether implemented policies or interventions over the years have influenced access times. The temporal stratification revealed that, despite occasional variations, there was no consistent improvement in adherence to the legislatively established deadlines during the analyzed period, as depicted in figures 3 and 4, which clearly illustrate the evolution or regression by DRS.

DRS: Regional Health Directorate.

Source: São Paulo Oncocenter Foundation Database. Prepared by the authors, 2025.

DRS: Regional Health Directorate.

Source: São Paulo Oncocenter Foundation Database. Prepared by the authors, 2025.

Regarding the qualitative analysis, which was substantiated by a documental and hermeneutic review of TCESP decisions over the studied period, revealing 186 decisive acts related to public health policy. Of these, 157 were categorized as instruments for the creation, organization, funding, and regulation of structures linked to the oncological health system, without addressing effective compliance with the deadlines established by the 60-day Law. The external control decisions predominantly demonstrated a concern with formal compliance, legality, and the economics of adjustments, based on the conducted review.

## DISCUSSION

The results reveal a persistent challenge in realizing the right to health, with gaps in adherence to legal deadlines and oversight performance. The quantitative findings of this study confirm previous research conclusions regarding the ongoing structural bottlenecks in complying with the 60-day Law.^(5)^ The fact that 46% of diagnoses and 56.4% of treatments commenced late between 2013 and 2024 reflects widespread systemic failure. This ineffective scenario has been consistently highlighted in the literature,^(22)^ as researchers have continuously raised concerns about the ongoing issues in oncological care, emphasizing the gap between legal prescriptions and actual practice within the system.

The observed regional disparities underscore the complexity of the healthcare network in São Paulo State. The triennial analysis reinforces that this issue has not shown consistent improvement over time. Delay in access, especially at the onset of treatment, is a critical factor, as the literature consistently correlates such delays in diagnosis and treatment initiation with significantly reduced chances of cure and poorer prognosis.^(4,23)^ Law No. 12,732/2012, by not stipulating direct and automatic administrative sanctions for public entities that fail to comply, tends to have its normative force diluted, effectively becoming a "mere piece of paper,"^(6,10)^ This may drive the judicialization of health as the main path to ensure this right, which, in turn, can overburden the Judiciary and ultimately distort the public policy itself.^(24)^

The effectiveness of the State in executing its core activities is essential for adequately addressing the needs of the Brazilian population, and the persistence of these delays contradicts the fundamental objectives of the 1988 Constitution.^(12)^ Moreover, TCESP's role is crucial in ensuring compliance with health legislation and overseeing the management of public resources. Thus, our qualitative analyses show that although the court exercises vigilant control over the legality and economy of matters, its decisions related to this topic have yet to address compliance with Law No. 12,732/12. Audit processes and accountability documents filed by the TCESP demonstrate concern about irregularities in physical and financial execution, as well as the clarity of goals and costs. However, there is a gap in evaluating the direct impact of these managements on care outcomes, particularly compliance with the deadlines set by the 60-day Law. The absence of objectively measurable goals in contracts and state health plans related to these deadlines complicates the external control's ability to induce accountability for effective outcomes.

TCESP's evaluation of results could contribute to inducing improvements in the effectiveness of public health policy.^(13,14)^ The lack of findings about waiting lists in measurable and monitorable goals by the TCESP exemplifies this fragility in accountability for compliance with the 60-day Law.

It is also noteworthy that outsourcing the management of essential services and the operation of CROSS by an OSS creates a strategic contradiction for the State, as delegating regulatory access to an entity that is already a SUS service provider may expose the system to a potential conflict of interest.^(9,25)^ The concern lies in the possibility that market dynamics or the internal priorities of the OSS, operating in dual capacity (regulator and provider), could influence patient direction and compliance with the deadlines established by the 60-day Law, distorting the principle of universality and comprehensiveness of the SUS.^(26,27)^

Our findings highlighted the need for a reorientation in oncology care policy management and the role of external oversight. Good governance and external oversight must transcend mere financial and administrative oversight, focusing on policy effectiveness and guaranteeing the fundamental right to adequate public service.^(9,28)^ Persistent delays in breast cancer diagnosis and treatment suggest that, while the existing regulatory framework and network structure are theoretically robust, significant challenges exist in their operationalization. The complexity of the SUS, with its regionalized and hierarchical structure, necessitates effective regulation to ensure comprehensive and equitable access.^(29,30)^

Furthermore, the analysis of the TCESP's actions, while demonstrating vigilance over the legality and economics of management contracts, reveals a predominant emphasis on formal aspects rather than on overseeing the effectiveness of results. The absence of clear and measurable goals, directly linked to access times in the oversight instruments and state plans analyzed, reduces the TCESP's ability to induce accountability and continuous improvement. Given this scenario, external oversight can:

Demand and oversee an explicit plan for complying with the 60-day Law, containing objective indicators per DRS for diagnosis time and treatment initiation, with continuous and public monitoring, alongside clear sanctions in cases of non-compliance.

Induce a review of the state cancer care plan focusing on effectiveness, to identify specific bottlenecks causing delays and propose strategies and investments aimed at overcoming these obstacles and ensuring the 60-day Law. This review may include process and outcome indicators to assess the real impact of actions on patients’ lives, aligning with the principles of good governance.^(14)^

Deepen the investigation of potential conflicts of interest with specific audits on the performance of OSS in access regulation and service provision, assessing whether referral decisions are optimized for legal deadline compliance or influenced by other factors, such as OSS capacity or the pursuit of higher profitability.

Expand transparency and accountability by requiring regular publication of data on waiting times, diagnosis, and treatment, enabling civil society and patients themselves to monitor system performance and advocate for improvements.

Only through more assertive and results-oriented actions can external oversight fulfill its constitutional role of guaranteeing the effectiveness of public policies, transforming the 60-day Law from an aspiration into a reality for breast cancer patients in the state of São Paulo.

## CONCLUSION

This study aimed to scrutinize the impact of Law No. 12,732/2012 on ensuring timely access to breast cancer diagnosis and treatment in São Paulo State between 2013 and 2024, alongside analyzing the role of external oversight mechanisms. The findings reveal a persistent and significant challenge in realizing the right to health within the state.

The quantitative analysis demonstrated widespread systemic failure, with 46% of diagnoses and 56.4% of treatments exceeding the legally stipulated deadlines. These delays were significant and persistent across all Regional Health Directorates, showing no consistent improvement throughout the analyzed period. Concurrently, the qualitative evaluation of the São Paulo State Court of Accounts (TCESP - *Tribunal de Contas do Estado de São Paulo*) decisions indicated a predominant focus on formal compliance and economic aspects rather than on evaluating the effectiveness of results, particularly concerning adherence to the 60-day Law. The analysis confirmed that the 60-day Law has had a limited impact in ensuring timely access to breast cancer diagnosis and treatment.

These results underscore the critical need for reorientation in oncology care policy management and a more assertive, results-oriented role for external oversight. It is imperative that external control strategically strengthens this public policy by inducing managerial accountability and guaranteeing the right to access and comprehensive care for patients. This includes demanding explicit compliance plans with measurable indicators, reviewing the state cancer care plan for effectiveness, and expanding transparency regarding waiting times and outcomes.

It is important to acknowledge that the quantitative analysis relied on secondary data, limiting in-depth exploration of individual-level causes for delays. Additionally, the qualitative analysis was confined to publicly available TCESP documents, which may not capture the full scope of internal deliberations within the oversight body.

## Data Availability

The content will be made available upon the article's publication.
